# Células Espumosas na Aterosclerose

**DOI:** 10.36660/abc.20220659

**Published:** 2022-10-05

**Authors:** Paulo Sampaio Gutierrez

**Affiliations:** 1 Hospital das Clínicas Faculdade de Medicina Universidade de São Paulo São Paulo SP Brasil Instituto do Coração do Hospital das Clínicas da Faculdade de Medicina da Universidade de São Paulo – Laboratório de Anatomia Patológica, São Paulo , SP – Brasil

**Keywords:** Aterosclerose, Patologia, Células Espumosas

**Figura 1:**
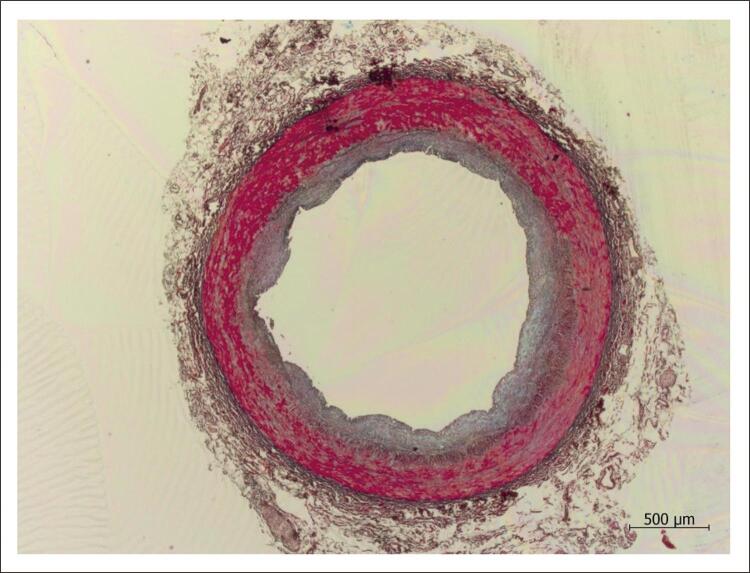
Corte histológico de artéria coronária humana normal, mostrando que na túnica íntima, ou seja, internamente à média (corada em vermelho), há tecido conjuntivo. Coloração pelo método pentacrômico de Movat; aumento da objetiva: 2,5x.

Já nas fases precoces das lesões ateroscleróticas há acúmulo de lipídeos na túnica íntima, os quais, nessa localização, sofrem modificações como oxidação e passam a ter a capacidade de induzir reações inflamatórias e consequentemente a progressão do processo patológico. Vale notar que há indícios de que interações com componentes presentes nessa camada arterial são importantes para que as gorduras sejam retidas e fiquem, portanto, sujeitas às referidas modificações que lhes trazem propriedades pró-inflamatórias. Nesse sentido, é interessante notar que, enquanto a maioria das espécies de mamíferos têm a íntima arterial “virtual” – ou seja, à microscopia de luz o endotélio parece repousar diretamente na lâmina elástica interna (ainda que a observação à microscopia eletrônica revele que não chega a ser assim) e sejam pouco susceptíveis à doença aterosclerótica, as espécies em que há quantidade de tecido que pode ser vista ao microscópio de luz separando o endotélio da lâmina elástica interna, como coelhos, porcos, símios e, principalmente, seres humanos ( Figura 1 ), são as que têm propensão a tal alteração.

**Figura 2:**
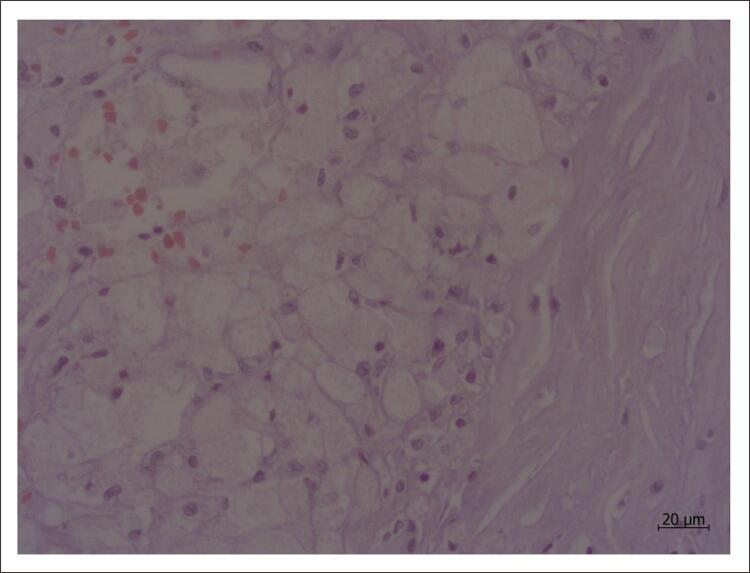
Corte histológico de artéria coronária humana apresentando células espumosas, caracterizadas por citoplasma com aspecto rendilhado. Coloração pelo método da hematoxilina & eosina; aumento da objetiva: 40x.

Ao serem retidos na íntima, os lipídeos se acumulam inicialmente no espaço extracelular, mas são também internalizados por células, que assumem ao microscópio o aspecto chamado de “células espumosas”. Isso acontece porque no processamento histológico habitual as amostras teciduais passam por uma série de banhos com a finalidade de desidratá-las e desengordurá-las, de modo de melhorar a qualidade dos cortes. Com a retirada da gordura, restam septos intracelulares que conferem aos citoplasmas padrão rendilhado, dando a impressão do que corresponderia em duas dimensões à vista de uma espuma ( Figura 2 ).

**Figura 3:**
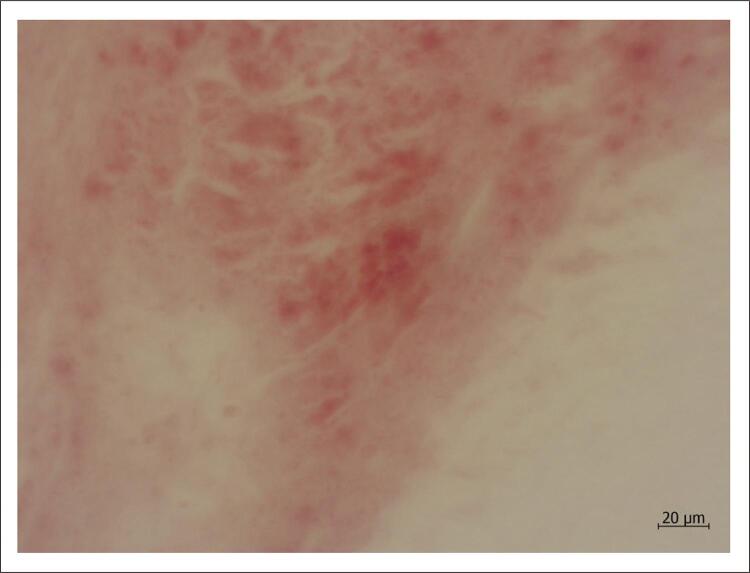
Corte histológico em amostra de artéria coronária humana submetida a congelação (e não ao processamento habitual) apresentando deposição de lípides, corados em vermelho. Devido à perda de qualidade no corte, é difícil precisar, mas ao menos parte da gordura parece estar localizada no espaço intracelular, possivelmente fazendo parte de células espumosas. Coloração pelo escarlate R; aumento da objetiva: 40x.

Há métodos de coloração para gordura sem o processamento habitual ( Figura 3 ), mas por outro lado perde-se qualidade no corte histológico. As mesmas células são também chamadas de “xantomatosas”, pelo grego “ξανθιά” (“xantiá”), que corresponde a “loiro, amarelo”; por ter grande quantidade de gordura, macroscopicamente a região tem essa cor. Grande parte das células espumosas é constituída por macrófagos, células que de fato têm como uma de suas funções primordiais internalizar material exógeno que apareça no interstício, seja qual for o órgão. Especificamente nas lesões ateroscleróticas, células musculares lisas e outras são também coadjuvantes nessa tarefa.

Castro et al. apresentam nesta edição dos *Arquivos Brasileiros de Cardiologia* um trabalho no qual estudam *in vitro* quais são os estímulos que levam à transformação de macrófagos em células espumosas; ^1^ como comentam, os macrófagos podem ter fenótipo classificado como M1, com alta expressão de proteínas pró-inflamatórias que podem contribuir para a formação da placa aterosclerótica, ou M2, que desempenham um papel preventivo, reduzindo o tamanho da placa e melhorando sua estabilidade.

Portanto, mais importante que apenas avaliar a formação da célula espumosa é verificar seu perfil pró-inflamatório. Para isso, analisaram como a formação das células espumosas contribui na produção de duas citocinas, o fator de necrose tumoral alfa (TNF-α) e a interleucina-6 (IL-6). Mostraram como concentrações de lipídeos de baixa densidade oxidados e tempo de incubação influenciam a formação dessas citocinas pró-inflamatórias, contribuindo assim para a elucidação de mecanismos celulares envolvidos na patogênese da aterosclerose.
