# Supplementary material for: Células Espumosas na Aterosclerose
Source: Arq Bras Cardiol. 2022 Oct 5;119(4):542–3. [Article in Portuguese] doi: 10.36660/abc.20220659 (PMC9563888; doi:10.36660/abc.20220659)
Supplement: Translation

# Translation: Foam Cells in Atherosclerosis

**Authors:** Paulo Sampaio Gutierrez

**Affiliations:** 1 Brazil Instituto do Coração do Hospital das Clínicas da Faculdade de Medicina da Universidade de São Paulo – Laboratório de Anatomia Patológica, São Paulo, SP – Brazil

**Keywords:** Atherosclerosis, Pathology, Foam Cells

In the early stages of atherosclerotic lesions, there is an accumulation of lipids in the tunica intima, which, in this location, undergo modifications such as oxidation and become capable of inducing inflammatory reactions and, consequently, the progression of the pathological process. It is worth noting that there are indications that interactions with components present in this arterial layer are important for fats to be retained and, therefore, subject to the modifications above that bring them pro-inflammatory properties. In this sense, it is interesting to note that while most mammalian species have a “virtual” arterial intima – that is, at light microscopy, the endothelium appears to lie directly on the internal elastic lamina (although observation electron microscopy reveals that this is not the case) and are less susceptible to atherosclerotic disease, species in which there is an amount of tissue that can be seen at light microscopy separating the endothelium from the internal elastic lamina, such as rabbits, pigs, apes and, mainly, human beings ( Figure 1 ), are the ones that are prone to such alteration.

**Figure 1:** Histological section of a normal human coronary artery shows that in the tunica intima, that is, internal to the media (stained red), there is connective tissue. Staining by Movat’s pentachrome method; objective magnification: 2.5x.

When retained in the intima, lipids initially accumulate in the extracellular space but are also internalized by cells, which assume the appearance called “foam cells” under the microscope. This is because, in the usual histological processing, tissue samples go through a series of baths to dehydrate and degrease them and improve the section quality. With the removal of the fat, intracellular septa are left that give the cytoplasm a lacy pattern, giving the impression of what would correspond in two dimensions to an appearance of foam ( figure 2 ). There are fat staining methods without the usual processing ( figure 3 ), but on the other hand, quality is lost in the histological section. The same cells are also called “xanthomatous,” by the Greek “ξανθιά” (“xanthia”), which corresponds to “blond, yellow”; because it has a large amount of fat, macroscopically, the region has this color. Most foam cells are made up of macrophages, cells that have as one of their primary functions to internalize exogenous material that appears in the interstitium, whatever the organ. Specifically, smooth muscle cells and others are also supportive in atherosclerotic lesions in this task.

**Figure 2:** Histological section of human coronary artery showing foam cells, characterized by cytoplasm with a lacy appearance. Staining by the hematoxylin & eosin method; objective magnification: 40x.

**Figure 3:** Histological section of a human coronary artery sample submitted to freezing (and not to the usual processing) shows lipid deposition, stained in red. Due to the loss of quality in the cut, it is difficult to be precise, but at least part of the fat appears to be located in the intracellular space, possibly forming part of foam cells. Scarlet coloration R; objective magnification: 40x.

Castro et al. present in this issue of *Arquivos Brasileiros de Cardiologia* a work in which they study in vitro the stimuli that lead to the transformation of macrophages into foam cells;^1^ as they comment, macrophages may have a phenotype classified as M1, with high expression of pro-inflammatory proteins that can contribute to the formation of atherosclerotic plaque, or M2, which play a preventive role, reducing the size of the plaque and improving its stability.

Therefore, verifying its pro-inflammatory profile is more important than just evaluating foam cell formation. For this, they analyzed how the formation of foam cells contributes to the production of two cytokines, tumor necrosis factor-alpha (TNF-α) and interleukin-6 (IL-6). They showed how concentrations of oxidized low-density lipids and incubation time influence the formation of these pro-inflammatory cytokines, thus contributing to the elucidation of cellular mechanisms involved in the pathogenesis of atherosclerosis.
